# Sleep disturbance and associated factors among Nigerian adults living with HIV in the dolutegravir era

**DOI:** 10.3389/frsle.2022.963529

**Published:** 2022-08-22

**Authors:** Adenekan O. Osiyemi, Eme Owoaje, Jennifer M. Mundt, Bibilola Oladeji, Oluwatosin Awolude, Adesola Ogunniyi, Prosper Okonkwo, Baiba Berzins, Babafemi O. Taiwo

**Affiliations:** ^1^Department of Family Medicine, University College Hospital, Ibadan, Nigeria; ^2^Department of Community Medicine, Faculty of Public Health, College of Medicine, University of Ibadan, Ibadan, Nigeria; ^3^Department of Neurology, Center for Circadian and Sleep Medicine, Northwestern University Feinberg School of Medicine, Chicago, IL, United States; ^4^Department of Psychiatry, University of Ibadan, Ibadan, Nigeria; ^5^Infectious Disease Institute, College of Medicine, University of Ibadan, Ibadan, Nigeria; ^6^Department of Medicine, College of Medicine, University of Ibadan, Ibadan, Nigeria; ^7^APIN Public Health Initiatives, Abuja, Nigeria; ^8^Division of Infectious Diseases, Northwestern University Feinberg School of Medicine, Chicago, IL, United States

**Keywords:** sleep, HIV, depression, anxiety, antiretroviral, Nigeria

## Abstract

Sleep disturbance is common among persons living with HIV (PLWH) causing significant health impacts. Nigeria recently switched from efavirenz to dolutegravir (DTG) for first-line antiretroviral therapy (ART). This study aimed to assess the prevalence of sleep disturbance and to determine factors associated with sleep disturbance among treatment-experienced PLWH. Using a cross-sectional study design and systematic random sampling, 300 participants were recruited from the Infectious Diseases Institute, Ibadan, Nigeria (IDI). Interviewer administered questionnaire was used to collect data regarding sociodemographic, sleep disturbance (Pittsburgh Sleep Quality Index; PSQI), depression (Patient Health Questionnaire; PHQ-9), anxiety (Generalized Anxiety Disorder-7; GAD-7), and psychoactive substance use. HIV-specific data were retrieved from IDI's medical records. The relationship between the PSQI score and the scores on the PHQ-9 and GAD-7 were explored with the Pearson correlation coefficient. Chi-square global tests of independence were used to assess factors associated with sleep disturbance and a multivariable binary logistic model was used to determine independent predictors of sleep disturbance. The mean age of the sample was 44.5 ± 11.4 (years), the average duration of HIV diagnosis was 8.13 ± 5.33 (years) and the majority were on DTG-based regimens (95%). Depression and anxiety were present in 14 and 17.3%, respectively. Prevalence of sleep disturbance (PSQI score of 6 or more) was 21.7%. Higher PSQI scores were associated with higher PHQ-9 scores (*r* = 0.526; *p* < 0.001) and higher GAD-7 scores (*r* = 0.529; *p* < 0.001). Sleep disturbance was associated with age (χ^2^ = 4.483, *p* = 0.038), marital status (χ^2^ = 7.187, *p* < 0.01), depression (χ^2^ = 46.589, *p* < 0.001), and anxiety (χ^2^ = 38.379, *p* < 0.001). There was no significant association between sleep disturbance and HIV clinical stage at diagnosis, virological suppression status, and ART regimen type (*p* > 0.05), whereas tea intake was associated with an absence of sleep disturbance (χ^2^ = 6.334, *p* < 0.014). Age (>45 years), depression and anxiety were associated with higher odds of sleep disturbance among PLWH. Sleep disturbance remains common among PLWH in the DTG era. Depression and anxiety are significant factors associated with sleep disturbance. Assessing these factors in future studies may improve the sleep health of PLWH.

## Introduction

Human immunodeficiency virus (HIV) infection and antiretroviral therapy (ART) can present with neurological complications. HIV persists in the central nervous system (CNS) because brain macrophages, microglial cells, and astrocytes serve as reservoirs (Minagar et al., 2008; King et al., 2013). The pathologically activated immune system associated with acute and chronic HIV infection disrupts sleep homeostasis (O'Brien et al., 2021). Due to sustained immune activity in the CNS, neurodegeneration of centers controlling sleep homeostasis could lead to disruption of sleep-wake physiology (O'Brien et al., 2021). The sleep architecture in people living with HIV (PLWH) is characterized by increased sleep onset latency, decreased sleep time, sleep fragmentation, and alteration of the slow wave sleep and rapid eye movement sleep patterns (Oshinaike et al., 2014; Tesoriero et al., 2019).

HIV infection and some ARTs have been associated with sleep disturbance (Minagar et al., 2008; Byun et al., 2016). Consequently, the prevalence of sleep disturbances in PLWH, reported as 29–97% (Reid and Dwyer, 2005; Taibi, 2012; Low et al., 2014; Awopeju et al., 2018) is remarkably higher than estimates of 3.9–40% found in the general population in low-income countries (Stranges et al., 2012). Furthermore, sleep disturbance among PLWH is under-recognized by health care providers and underreported by patients (Desalu et al., 2018) just as the prevalence of poor sleep quality is between 20 and 59% (Oshinaike et al., 2014; Shittu et al., 2015; Yunusa et al., 2016; Adeoti, 2018; Awopeju et al., 2018; Desalu et al., 2018). Consequently, underreporting and inadequate recognition of sleep disturbance among PLWH may significantly worsen the quality of life.

Risk factors for sleep problems among PLWH have been examined in some studies of which socio-demographic contributors include older age, female gender, and employment status (Estrada et al., 2018). Some HIV-specific factors linked with sleep disturbance include duration of HIV diagnosis and treatment, delayed initiation of treatment and suboptimal adherence, concomitant medication use, and virological non-suppression (Estrada et al., 2018). Other risk factors include adverse effects of ARTs [e.g., efavirenz or dolutegravir (DTG)], mental disorders and substance abuse (Oshinaike et al., 2014; Wu et al., 2015).

In 2018, 2 years after recommending DTG-based ART as an alternative regimen, the World Health Organization (WHO) elevated DTG-based ART to preferred status (World Health Organization, 2019). Uganda and Nigeria were early DTG adopters in Africa with Nigeria initiating a successful transition from efavirenz, a drug associated with sleep disturbances, to DTG-based HIV treatment in 2017^1^ (Black and Schwartz, 2018). A pilot study to examine patient experiences in both countries revealed sleep disturbances (8%) and increased appetite (17%) were the commonest DTG side effects among patients in Uganda and Nigeria, respectively (Campbell et al., 2018). Since the widespread rollout of DTG as first-line ART for Nigerian adults, no study has examined sleep disturbance among PLWH on treatment though discontinuation of DTG-based ART due to neuropsychiatric adverse events such as sleep disturbance is around 3.5% (Hoffmann and Llibre, 2019). This study aimed to assess the prevalence of sleep disturbance and to determine factors associated with sleep disturbance among treatment-experienced PLWH.

## Materials and methods

### Participants and procedures

This study was conducted at the adult HIV clinic of the Infectious Disease Institute, College of Medicine, University of Ibadan (IDI/CoMUI), which receives support from the CDC-funded AIDS Prevention Initiative in Nigeria (APIN) Public Health Initiatives. The IDI/CoMUI provides HIV care services to more than 10,000 adults. Ibadan is the third largest Nigerian city with a mid-size urban environment. The study site which belongs to a university-based tertiary health care system, provides HIV care services to a large catchment area covering the southwestern region of Nigeria. Using a cross-sectional study design, we recruited participants at IDI/CoMUI from June 1st till July 31st, 2020. Individuals aged 18 years and above, diagnosed with HIV, who had been on ART for at least 6 months were consented and enrolled. Exclusion criteria included being newly diagnosed or ART-naive, inability or failure to provide consent, current CNS infection or other debilitating medical illness, current diagnosis of a sleep disorder (insomnia, narcolepsy, obstructive sleep apnea, restless leg syndrome), current diagnosis of a mental disorder, or use of efavirenz-based ART.

The sample size was determined using the formula for prevalence studies based on the prevalence of sleep disturbance reported by Awopeju et al. (2018) and power considerations for cross-sectional studies (Lu et al., 2013). A minimum sample size of 286 patients was to be recruited for this study. With an anticipated 5% missing data, a sample size of 300 patients was proposed. Patients were selected by systematic random sampling. From the medical records, approximately 800 adults who received care each week at the study site met the inclusion criteria. Based on this number, it was estimated that 6,400 eligible patients would be expected to attend the clinic within 2 months (8 weeks) of the study period. The sampling interval of 21 was calculated by dividing the estimated total number of clinic attendees during the study period (6,400) by the sample size (300). At least nine eligible participants were recruited each clinic day until the required sample size was attained. Research staff and/or the clinic head nurse made announcements about the study at the daily health talks that precedes clinic activities for the day.

### Measures

#### Socio-demographic characteristics

Factors considered included age, gender, level of education, marital status, employment status, and monthly wage which was grouped into two; less than the monthly minimum wage in Nigeria as of June 2019 (30,000 Naira/approximately $97) and minimum wage or more.

#### HIV-specific characteristics

These included duration of HIV diagnosis, HIV WHO clinical stage (Federal Ministry of Health, National AIDS, Control Program, 2016) at diagnosis, HIV viral load, date of ART initiation, the interval between diagnosis and initiation of ART, and being on DTG- based ART regimen or not. CD4+ count was not included because this was not routinely collected at the study site for treatment-experienced patients at the time of this study was conducted.

#### Sleep disturbance measure

The Pittsburgh Sleep Quality Index (PSQI) was used to identify sleep disturbances (Oshinaike et al., 2014). PSQI is a 19-item questionnaire that assesses seven sleep components including sleep quality, sleep latency, sleep duration, habitual sleep efficiency, sleep disturbances, use of hypnotics, and daytime dysfunction during the last month. Each component is scored from zero to three, with the total score on the instrument ranging between 0 and 21. PSQI has adequate validity with laboratory polysomnography (Brandolim Becker et al., 2018). For this study, sleep disturbance was defined as present if a participant had a PSQI score of 6 or more.

#### Mental health measures

Anxiety was assessed using the General Anxiety Disorder-7 (GAD-7). Anxiety was present where participants scored ≥5. Depression was assessed with the Patient Health Questionnaire (PHQ-9). A score of ≥5 implied the presence of depression. GAD-7 and PHQ-9 have good psychometric properties (Kroenke et al., 2001; Rutter and Brown, 2017) and have been validated for use among PLWH (Allavena et al., 2016; Yunusa et al., 2016; Adeoti, 2018).

#### Psychoactive substance history

Participants indicated yes or no to six questions relating to the intake of six psychoactive substances in the past month namely alcohol, coffee, tea, cigarette, and cannabis.

### Data collection instrument

Data were collected using an interviewer-administered questionnaire. The questionnaire was translated to the Yoruba language (the indigenous language at the study site) and back-translated to the English language for retention of the original meaning and easy administration of the questions. The data collection tool was pretested among 30 respondents at the adult HIV Clinic of Adeoyo Maternity Teaching Hospital, Adeoyo, Ibadan. The findings from this pretesting process were used to make necessary adjustments to the questionnaire. The questionnaire contained socio-demographic data, measures of sleep disturbance, psychoactive substance use, anxiety and depression. HIV-related details were retrieved from IDI's medical records with the permission of the APIN Public Health Initiatives. The University of Ibadan/University College Hospital Institutional Review Board approved the protocol for the study.

### Ethical consideration

Ethical approval was obtained from the Health Research Ethics Committee of the University of Ibadan. IRB assigned number UI/EC/20/0039 and APIN IRB, reference number IRB 048–FR. Informed verbal and written consent was obtained from each participant and they were assured of anonymity and confidentiality by assigning numerical codes to each questionnaire.

### Data analysis

Descriptive statistics were presented in frequency tables. Pearson correlation coefficient was used to explore the relationship between the PSQI score and the scores on the PHQ-9 and GAD-7 for depression and anxiety, respectively. Chi-square global tests of independence were used to assess factors associated with sleep disturbance. Variables that were significant at *p* < 0.05 level in the chi-square tests were retained in the multivariable binary logistic model to determine independent predictors of sleep disturbance. Confidence intervals and significance levels were set at 95 and 5%, respectively. Data were analyzed using SPSS software version 21.

## Results

The mean age of the participants (*N* = 300) was 44.5 ± 11.4 (years); females were 76.7%, 64% were married, and 87.7% were employed. The average duration of HIV diagnosis was 8.13 ± 5.33 (years). The majority (82%) had virological suppression (plasma HIV-1 RNA <200 copies/mL) and were on DTG-based regimens (95%) whereas depression and anxiety were present in 14 and 17.3%, respectively. The mean PSQI score was 4.0 ± 2.9 prevalence of sleep disturbance (PSQI score ≥ 6) in this study was 21.7%. No participant reported smoking cigarettes or cannabis. Other descriptive details of the study are presented in Table 1.

**Table 1:** Socio-demographic characteristics and other descriptive factors of study participants.

**Variable**	**Frequency**	**Percentage**
**Gender**		
Male	70	23.3
Female	230	76.7
**Age (years)**		
<30	24	8
30–39	63	21
40–49	118	39.3
50–59	66	22
≥60	29	9.7
**Marital status**		
Unmarried	118	39.3
Married	182	60.7
**Level of education**		
Secondary or less	192	64
Post-secondary	108	36
**Employment status**		
Unemployed	37	12.3
Employed	263	87.7
**Monthly income**		
Less than minimum wage	197	65.7
Minimum wage	103	34.3
HIV stage at diagnosis^*^		
1	89	29.7
2	67	22.3
3	68	7.7
4	23	17.7
**Time difference between diagnosis and initiation of ART**		
Within 1 month of diagnosis	220	73.3
>1 month	80	26.7
**Virologically suppressed (plasma HIV-1 RNA** **<** **200 copies/mL)**		
Yes	246	82
No	54	18
**ART regimen**		
Dolutegravir-based	285	95
Non-dolutegravir-based	15	5
**Depression**		
Yes	42	14
No	258	86
**Anxiety**		
Yes	52	17.3
No	248	82.7
**Alcohol use**		
Yes	34	11.3
No	266	88.7
**Coffee intake**		
Yes	26	8.7
No	274	91.3
**Tea intake**		
Yes	213	71
No	87	29
**Sleep disturbance**		
Yes	65	21.7
No	235	78.3

* *Fifty three (17.7%) participants had no record of HIV clinical stage at the time of HIV diagnosis*.

### Factors associated with sleep disturbance

Higher PHQ-9 scores were associated with higher PSQI scores (*r* = 0.526; *p* < 0.001) just as higher GAD-7 scores were associated with higher PSQI scores (*r* = 0.529; *p* < 0.001). As shown in Table 2, age and marital status were associated with sleep disturbance. Moreover, depression (χ^2^ = 46.589, *p* < 0.001) and anxiety (χ^2^ = 38.379, *p* < 0.001) were associated with sleep disturbance. There was no significant association between sleep disturbance and HIV-related factors such as clinical stage at diagnosis, virological suppression status, and ART regimen (*p* > 0.05), whereas tea intake was associated with the absence of sleep disturbance (χ^2^ <6.334, *p* = 0.014).

**Table 2:** Sleep disturbance and its associated factors using Chi square test.

**Variable**	**Sleep disturbance**		***p*-value**
	**Yes (%)**	**No (%)**	
**Gender**	
Male	18 (27.7)	52 (22.1)	0.407
Female	47 (72.3)	183 (77.9)	
**Age (years)^a^**	46.8 ± 10.7	43.8 ± 11.5	0.066
**Marital status**	
Unmarried	35 (53.8)	83 (35.3)	0.007
Married	30 (46.2)	152 (64.7)	
**Level of education**	
Secondary or less	36 (55.4)	156 (66.4)	0.102
Post-secondary	29 (44.6)	79 (33.6)	
**Employment status**	
Unemployed	10 (15.4)	27 (11.5)	0.398
Employed	55 (84.6)	208 (88.5)	
**Monthly income**	
Less than minimum wage	38 (58.5)	159 (67.7)	0.185
Minimum wage	27 (41.5)	76 (32.3)	
**HIV stage at diagnosis**	
1	16 (31.4)	73 (37.2)	0.31
2	12 (23.5)	55 (28.1)	
3	15 (29.4)	53 (27)	
4	8 (15.7)	15 (7.7)	
**Time difference between diagnosis and initiation of ART**		
Within 1 month of diagnosis	46 (70.8)	174 (74)	0.597
>1 month	19 (29.2)	61 (26)	
**Virologically suppressed (plasma HIV-1 RNA** **<** **200 copies/mL)**		
Yes	49 (75.4)	197 (83.8)	0.117
No	16 (24.6)	38 (16.2)	
**ART regimen**		
Dolutegravir-based	61 (93.8)	224 (95.3)	0.630
Non-dolutegravir-based	4 (6.2)	11 (4.7)	
**Depression**		
Yes	26 (40)	16 (6.8)	<0.001
No	39 (60)	219 (93.2)	
**Depression scores ^a^**	4.6 ± 5.5	1.2 ± 2.5	<0.001
**Anxiety**	
Yes	28 (43.1)	24 (10.2)	<0.001
No	37 (56.9)	211 (89.8)	
**Anxiety score ^a^**	5.5 ± 5.6	1.5 ± 2.8	<0.001
**Alcohol intake**		
Yes	11 (16.9)	23 (9.8)	0.108
No	54 (83.1)	212 (90.2)	
**Coffee intake**		
Yes	9 (13.8)	17 (7.2)	0.094
No	56 (86.2)	218 (92.8)	
**Tea intake**		
Yes	38 (58.5)	175 (74.5)	0.012
No	27 (41.5)	60 (25.5)	

a *t-test*.

The multivariate logistic regression analysis showed that independent predictors of sleep disturbance were age, depression, and anxiety as shown in Table 3. For example, participants with anxiety (GAD ≥ 5) had a three-fold higher odds of sleep disturbance than those with no anxiety (GAD <5) (odds ratio [OR], 2.88; 95% CI, 1.25–6.62; *p* = 0.013).

**Table 3:** Independent predictors of sleep disturbance.

**Factor**	**Adjusted odds ratio (95% Confidence interval)**	***p*-value**
**Age (years)**
>45	2.45 (1.28–4.67)	0.007
≤ 45	1	
**Marital Status**
Married	0.68 (0.36–1.27)	0.235
Unmarried	1	
**Depression**
Present	5.12 (2.20–11.94)	<0.001
Absent	1	
**Anxiety**
Present	3.24 (1.48–7.10)	0.003
Absent	1	
**Tea intake**
Yes	0.73 (0.37–1.46)	0.380
No		

## Discussion

This study aimed to assess the prevalence of sleep disturbance and to determine factors associated with sleep disturbance among treatment-experienced PLWH following widespread DTG roll-out in Nigeria. Using the PSQI, the prevalence of sleep disturbance (21%) in this study mirrors that the findings of Awopeju et al. (2018) (24%) and Desalu et al. (2018) (25%). Oshinaike's study reported sleep disturbance prevalence of 59.3% (Oshinaike et al., 2014) although participants were on efavirenz which is known to cause sleep problems. In other reports by Adeoti et al. as well as Huang et al., high prevalence rates of sleep disturbance (43%) were observed (Huang et al., 2017; Adeoti, 2018). The variation in prevalence may be attributable to differences in measurement tools, study periods and study sites. Our study in Nigeria confirms that sleep problems in PLWH are more common when compared with the general adult population, as reported in Indonesia and Kenya (Stranges et al., 2012). These results reinforce the public health significance of sleep disorders in PLWH, emphasizing the need for further research to design and implement interventions.

We found a high burden of undiagnosed anxiety (17.3%) and depression (14%) among PLWH, similar to earlier reports (Shittu et al., 2015; Allavena et al., 2016; Yunusa et al., 2016; Estrada et al., 2018). In these earlier reports, the prevalence of anxiety and depression ranges between 7.6 and 42%. We note that with exclusion of participants with pre-existing mood and sleep disorders, the prevalence of these conditions in this study may be underestimates. Anxiety and depression moderate sleep problems in the general population and among PLWH (Allavena et al., 2016).

We report a direct relationship between PSQI scores and scores on mood disorder scales (PHQ 9 and GAD 7). Junqueira et al. (2008) had described similar relationship among women living with HIV. Adeoti (2018) reported the odds of sleep problem as 2.5 times higher among depressed PLWH and Allavena et al. (2016) observed even higher odds of 4.6 in similar population. After adjusting for socio-demographic variables, the odds of having sleep disturbance were significantly higher among participants who reported either anxiety or depression. This is the strongest association found in our study and is consistent with other studies. Depression and anxiety, which negatively impact the quality of life (Deshmukh et al., 2017) of PLWH, have bidirectional relationships with sleep problems (Gutierrez et al., 2019). With easy-to-use, validated mental health screening tools as done in this study (Satre et al., 2019), it has become easier to identify depression and anxiety in routine clinical care. Translating this research finding into practice, especially in low resource settings is recommended. Furthermore, incorporating cognitive and behavioral strategies that address depression and anxiety (Spies et al., 2013; Satre et al., 2019) into clinical practice may mitigate the contributions of these co-morbidities to sleep problems among PLWH, and addressing sleep health may improve mood disorders due to the reciprocal relationship between these conditions.

While some reports (Oshinaike et al., 2014; Adeoti, 2018; Desalu et al., 2018) have indicated a link between poor sleep and certain HIV-related factors such as duration of HIV diagnosis and the type of ART, our study did not show any significant association between these HIV-related factors and sleep disturbance. Our findings mirror reports of Huang et al. (2017). Regarding sleep disturbance in the DTG era, to our knowledge, our study is the first to assess sleep-related problems among PLWH in Africa since the widespread roll-out of DTG-based ART. While most participants (95%) were on a DTG -based regimen which has been linked to poor sleep in some PLWH (Hoffmann and Llibre, 2019), we did not find an association in this study. The duration for which the participants had been on the medication may explain why they did not report poor sleep. The average duration of HIV diagnosis in this study was 8 years indicating participants might have been on DTG-based ART for a fair period given that the drug was nationally adopted in 2017^1^ (World Health Organization, 2019). While it is possible that our study could have captured early cases of DTG discontinuation due to insomnia if it had been conducted in the early period of DTG rollout, it is also possible that DTG may not be a significant driver of insomnia and other psychiatric morbidities, as reported by other investigators (Hsu et al., 2018). Nevertheless, our results agree with other studies that the effects of DTG on sleep are less after prolonged use (Elzi et al., 2017; Capetti et al., 2018; Elliot et al., 2019).

Sleep disturbances occur throughout all clinical stages of HIV infection, being more prevalent in the advanced stage (Reid and Dwyer, 2005; Oshinaike et al., 2014). Despite over half of the sample population being in early HIV clinical stages at diagnosis in our study, that they were virologically suppressed at the time of data collection, may explain the absence of an association between sleep disturbance and duration of HIV diagnosis and virological suppression status. Furthermore, with the average duration of HIV diagnosis being 8 years, it is possible respondents had been on ARTs for a while which could limit the negative effects of uncontrolled HIV on sleep. While it has been reported that HIV and antiretrovirals may cause sleep disturbance, the regimen and medication adherence could have attenuated these negative factors in this study.

No participant reported smoking. This may indicate some social desirability bias among respondents, since pooled prevalence of smoking in Nigeria is 10.4% and tobacco use is common among PLWH (Mdege et al., 2017; Adeloye et al., 2019; Murphy et al., 2019). Furthermore, of the psychostimulants examined in this study, tea intake was associated with the absence of poor sleep. This finding may be due to a limitation of the study questionnaire, which did not ascertain the specific types of tea (black, green, herbal, etc.) consumed by participants. Non-caffeinated varieties such as chamomile have been linked to relaxation and improved sleep among adults (Chang and Chen, 2016), whereas caffeinated teas have a dose-dependent disruptive effect on sleep (Hindmarch et al., 2000). Certain types of tea consumption may bear some benefit in our sample and further interventional research could consider this beverage in modifying sleep among PLWH. We note that the association of tea was only present in chi square test. After adjusting for other factors, this beverage did not independently reduce the odds of having sleep disturbance, implying sleep problems among PLWH may be explained by factors asides from tea, such as mood disorders and older age.

Our study had some strengths. The validated measures used are pragmatic for routine clinical settings since these tools are inexpensive and brief. This study also indicates that DTG -based regimen may not be associated with sleep problems in the long run. This is reassuring given the advantages of DTG-based regimens such as high potency and high barrier for resistance.

A limitation of this study is the cross-sectional nature which did not allow for proper evaluation of whether the sleep disturbances were transient or chronic, nor can we conclude causality between sleep disturbance and the highlighted factors. This is was a single center study where CD4+ counts were not routinely assessed among treatment experienced PLWH. In addition, the PSQI lacks specificity for insomnia. We also did not assess pain in the study sample and pain has been linked to sleep disturbance. Furthermore, this study did not characterize the types of tea consumed by participants, so further inferences could not be made regarding the effect of tea consumption on sleep among PLWH.

## Conclusion

Sleep disturbance remains common among PLWH in Nigeria in the DTG era but is not associated with DTG use. Depression and anxiety are the significant factors associated with sleep disturbance and as such, addressing these factors may improve the sleep health of PLWH. With a bidirectional relationship between sleep problems and mood disorders, treatment of sleep disturbance may also mitigate anxiety and depression among PLWH.

## Data availability statement

The raw data supporting the conclusions of this article will be made available by the authors, without undue reservation.

## Ethics statement

The studies involving human participants were reviewed and approved by Health Research Ethics Committee of the University of Ibadan. IRB assigned number UI/EC/20/0039 and APIN IRB, reference number IRB 048–FR. The patients/participants provided their written informed consent to participate in this study.

## Author contributions

AOO was the principal investigator for the study. AOO, EO, JM, BO, BT, and BB contributed to this research's conceptualization, design, analysis, and reporting. AO, OA, and PO contributed to the ethical considerations of the study. AOO, EO, JM, BB, and BT contributed to all aspects of the article, including editing and proofreading the article. All authors approved the submitted version.

## Funding

Research training for this publication was supported by the Fogarty International Center of the National Institutes of Health and National Institute of Mental Health of the National Institutes of Health under Award Number D43TW009608.

## Conflict of interest

The authors declare that the research was conducted in the absence of any commercial or financial relationships that could be construed as a potential conflict of interest.

## Publisher's note

All claims expressed in this article are solely those of the authors and do not necessarily represent those of their affiliated organizations, or those of the publisher, the editors and the reviewers. Any product that may be evaluated in this article, or claim that may be made by its manufacturer, is not guaranteed or endorsed by the publisher.

## Author disclaimer

The content is solely the responsibility of the authors and does not necessarily represent the official views of the National Institutes of Health.

## Footnotes

^1^Dolutegravir-based ART recommended for all - if reliable contraception is available | aidsmap.
